# *Bordetella pertussis, Chlamydia pneumoniae, and Mycoplasma pneumoniae* Findings in Children During COVID-19 Pandemic in Finland

**DOI:** 10.1007/s42399-022-01251-9

**Published:** 2022-07-14

**Authors:** Ilari Kuitunen

**Affiliations:** 1 Institute of Clinical Medicine and Department of Pediatrics, University of Eastern Finland, Kuopio, Finland; 2 Department of Pediatrics, Mikkeli Central Hospital, 35-37, 50100 Mikkeli, Finland

**Keywords:** Epidemiology, Surveillance, Bordetella pertussis, Chlamydia pneumoniae, Mycoplasma pneumoniae

## Abstract

Social restrictions effectively interrupted the typical respiratory virus circulation. The aim of this report is to describe how atypical pneumonia bacteria detections have changed during the COVID-19 pandemic in Finnish children. *Bordetella pertussis*,* Chlamydia pneumoniae*, and* Mycoplasma pneumoniae* findings were gathered from the National Infectious Diseases Register from 2015 to 2021 and stratified into three age groups (0–4, 5–9, and 10–14 years). Incidences per 100,000 children were calculated and 2020 and 2021 was compared to the mean incidence of reference years (2015–2019) by incidence rate ratios. *B. pertussis* incidence was 32% lower in 2020 and 88% lower in 2021 compared to reference years. *C. pneumoniae d*etections decreased rapidly in 2020 and not a single detection of *C. pneumoniae* was reported to the register in 2021. *M. pneumoniae* incidence was 72% lower in 2020 and 89% lower in 2021 than in the reference years. All these findings were seen in all age groups. As the incidences of *B. pertussis*,* C. pneumoniae*, and *M. pneumoniae* detections have been low throughout the pandemic, it seems that the social restrictions have been effective in preventing the spreading of these respiratory bacteria in children.

**Supplementary Information:**

The online version contains supplementary material available at 10.1007/s42399-022-01251-9.

## Introduction

Social restriction set against COVID-19 in March 2020 interrupted the circulation of respiratory pathogens in Finland [1, 2]. Schools and day care facilities were then opened in May 2020 and have remained since open in Finland. Social restrictions set towards adults prevented Influenza and respiratory syncytial virus seasons in Finland in Winter 2020–2021 but did not prevent the spreading of rhinoviruses, which continued normally [3–5]. After the last restrictions were cleared mostly in August 2021, Finland had record high parainfluenza epidemic [6]. Majority of the published literature has focused on the epidemiology and incidences of viral respiratory infections during the COVID-19 pandemic.

A previous study from England reported a 98% reduction in *Bordetella pertussis* detections among infants (age less than 1 years) [7]. A study from Israeli found that the rates of *B. pertussis* and *Mycoplasma pneumoniae* decreased in hospitalized patients with respiratory tract infections during the early months of the pandemic. [8] Two Chinese studies reported major decreases in the *Mycoplasma pneumoniae* detections rates in Children during the pandemic [9, 10]. A large global study found also that the incidence of *M. pneumoniae* detections decreased especially with direct tests (polymerase chain reaction PCR), whereas the antibody detections did not decrease [11]. *Chlamydia pneumoniae* incidence has not been assessed in children during the pandemic.

The aim of this manuscript is to report the incidence of laboratory confirmed cases of *B. pertussis*,* C. pneumoniae*,* and M. pneumoniae* in Finnish children during the COVID-19 pandemic.

## Methods

All laboratory confirmed findings of *B. pertussis*,* C. pneumoniae*, *and M. pneumoniae* were included from the National Infectious Disease Register of Finland. All diagnostic laboratories are mandated by the Law of Contagious Diseases to report all positive findings of notifiable diseases. Therefore, the coverage of the register is excellent [12]. As the register does not contain information on the number of tests performed, the estimation on the COVID-19 pandemic to the testing numbers was estimated based on the single regional laboratory data. Southern Savonia heathcare region and laboratory produces testing for a population of 100,000 (of those children approximately 13,000). For this study information on antibody tests performed from 2015 to 2021 were included.

*B. pertussis*,* C. pneumoniae*, and* M. pneumoniae* are tested in Finland as direct way as those part of the multiplex PCR panels. These samples are nasopharyngeal swabs and taken typically in acute respiratory infections and this test is typically used only in hospitalized patients in pediatric wards. The register also contains information on indirect findings (typically IgM positive serology and/or clear increase in IgG serology during two to four weeks). In cases of for example prolonged cough are these three pathogens actively searched in every patient with antibody tests. Prolonged cough is defined as cough lasting at least 6 to 8 weeks in Finland and then the examination of these three pathogens is part of the standard protocol.

Children aged 0–14 years were included and stratified into three age groups (0–4, 5–9, and 10–14 years) based on default stratification in the register. Study period was from January 2015 to December 2021. Mean yearly incidence per 100,000 children was calculated for the reference years (2015–2019) and compared to 2020 and 2021 by incidence rate ratios (IRR) with 95% confidence intervals (CI). The number of children in each age group was gathered from Population Information Statistics [13]. This study did not need ethical committee evaluation due to the register-based retrospective study design. This study has the research permission from the Southern Savonia healthcare officials to gain access to laboratory testing data.

## Results

**Fig. 1:**
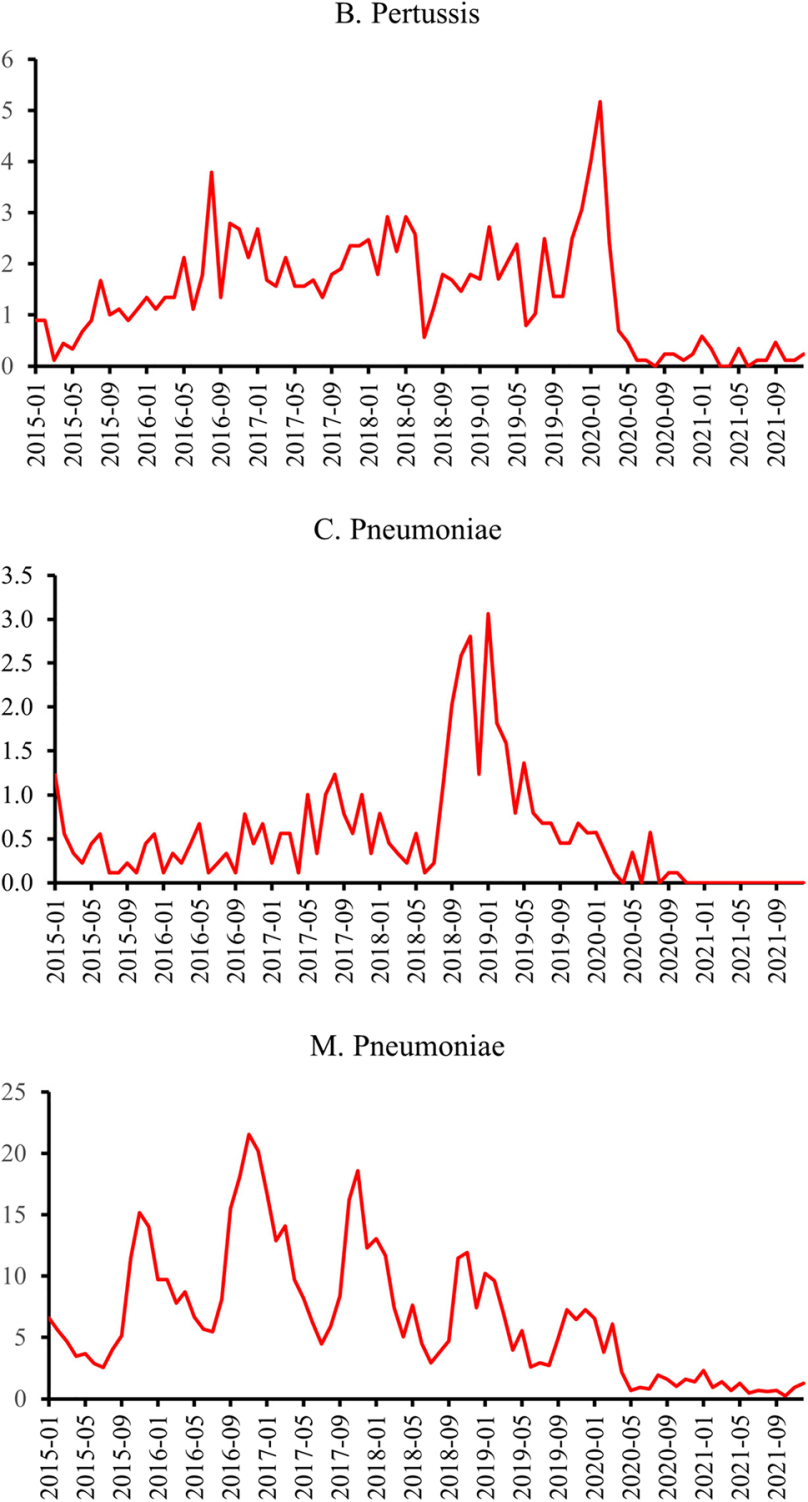
Monthly incidence per 100,000 of *B. pertussis*,* C. pneumoniae*, and *M. pneumoniae* detections among children aged 0–14 years in Finland from January 2015 to December 2021

A total of 1050 *B. pertussis*, 397 *C. pneumoniae*, and 4922 *M. pneumoniae* detections were included from the register. The monthly incidence of *B. pertussis* decreased rapidly in the March 2020 and have remained low since to the end of 2021 (Figure 1). Incidence was 32% lower in 2020 and 88% lower in 2021 compared to reference years (Table 1). The most prominent decrease was observed among children aged 10–14 years in 2021 (IRR 0.03 CI 0.01–0.11).

**Table 1:** Yearly number of detections and incidence in 2020 and 2021 compared to mean yearly incidence of reference years (2015–2019) by incidence rate ratios (IRR) with 95% confidence intervals

	2015–2019		2020				2021			
	*N*	Incidence	N	Incidence		IRR (95% CI)	*N*	Incidence	IRR (95% CI)	
*Bordetella pertussis*										
Total	909	20.4	120	13.8		0.68 (0.56–0.82)	21	2.4	0.12 (0.08–0.18)	
0–4 years	286	13.3	32	9.5		0.62 (0.43–0.90)	16	6.5	0.32 (0.20–0.54)	
5–9 years	234	28.2	29	19.1		0.71 (0.48–1.05)	2	0.7	0.05 (0.01–0.20)	
10–14 years	482	20.4	59	13.8		0.68 (0.52–0.89)	3	1.0	0.03 (0.01–0.11)	
*Chlamydia pneumoniae*										
Total	397	8.5	19	2.2		0.26 (0.16–0.41)	0	0.0	0.0 (N/A–N/A)	
0–4 years	26	1.8	1	0.4		0.22 (0.03–1.65)	0	0.0	0.0 (N/A–N/A)	
5–9 years	131	8.4	3	1.0		0.12 (0.04–0.37)	0	0.0	0.0 (N/A–N/A)	
10–14 years	240	15.1	15	4.9		0.32 (0.19–0.54)	0	0.0	0.0 (N/A–N/A)	
*Mycoplasma pneumoniae*										
Total	4 574	102.6	249	28.6		0.28 (0.25–0.35)	99	11.5	0.11 (0.09–0.14)	
0–4 years	512	35.8	36	14.1		0.39 (0.28–0.55)	12	2.9	0.14 (0.08–0.24)	
5–9 years	1 702	111.3	94	30.7		0.28 (0.22–0.34)	32	10.6	0.10 (0.07–0.14)	
10–14 years	2 360	158.2	119	38.5		0.24 (0.20–0.29)	55	17.6	0.11 (0.09–0.15)	

*C. pneumoniae* incidence has been previously relatively low, but before the pandemic clear epidemic peak occurred in winter 2018–2019 (Figure 1). Detections decreased rapidly in 2020 and not a single detection of *C. pneumoniae* was reported to the register in 2021 (Table 1).

The monthly incidence of *M. pneumoniae* has had relatively predictable trends prior to pandemic (Figure 1). During the pandemic the monthly incidence has remained notably lower than in the reference years. The yearly incidence was 72% lower in 2020 and 89% lower in 2021 than in the reference years (Table 1).

Yearly number of tests decreased for *M. pneumoniae* and *B. pertussis* (Table 2). The decrease was more prominent in the first pandemic year than in the second. The number of *C. pneumoniae* antibody tests increased by 47.2 % in 2020 and 80.4% in 2021 compared to the mean of five pre-pandemic years.

**Table 2:** Yearly number of *Bordetella pertussis*,* Chlamydia pneumoniae*, and *Mycoplasma pneumoniae* antibody tests in Southern Savonia healthcare region. Pandemic years (2020 and 2021) compared to mean of five pre-pandemic years (2015 to 2019)

	2015–2019	2020		2021	
	*N*	*N*	Change	*N*	Change
*Bordetella pertussis*	138	44	− 62.1%	61	− 55.8%
*Chlamydia pneumoniae*	89	43	− 51.7%	60	− 32.6%
*Mycoplasma pneumoniae*	36	53	+ 47.2%	65	+ 80.6%

## Discussion

The incidences of *B. pertussis*,* C. pneumoniae*, and *M. pneumoniae* have been notably lower during the COVID-19 pandemic in all aged children. Social restrictions and improved hand hygiene measures have most likely decreased the incidences of these respiratory bacteria. The decrease began right after the lockdown began in March 2020. Although the restrictions set towards children were partially eased already in May 2020 and children have attended to schools and day cares normally without masks since August 2020, the respiratory bacterial pathogen findings have remained in low levels.

The observed decrease in *B. pertussis* was 88% in our study, whereas the English study presented a decrease of 97%, but the detection rates in this study were relatively rare (only 21 detections in 2021), so these findings were practically similar [7]. The results of this study are also similar to the previous reports regarding the decrease in the detections of *M. pneumoniae* [8–11]. There were no previous studies focusing on *C. pneumoniae*. But the detection rate in Finnish children has been practically zero throughout the pandemic. Some reports have assessed possible co-infections in COVID-19 patients and *C. pneumoniae* has been found in adult-aged COVID-19 patients [14]. A Brazilian study tested patients with suspected COVID-19 by multiplex panels and found cases of *C. pneumoniae* in non-COVID-19 patients [15].

The main limitation of this study is the lack of testing numbers, as these are not reported to the National Infectious Disease Register. To overcome this issue, a subsample of one regional laboratory was analyzed which showed that although the testing rates were lower than previously, the testing continued. However, the reduced testing numbers could have been due to reduced disease burden instead of limited testing capacity. Thus, must be noted that the primary care visit rate returned to normal in Finland in August 2020 after being lower during the initial lockdown from March 2020 to June 2020, and the visit rate has remained normal since [16, 17]. The reason for the increased *C. pneumoniae* testing remains unknown and is interesting as the nationwide detections rate have been zero during the second pandemic year. Further limitation is that the register does not provide information on re-infections or co-infections as it records only the microbial findings per microbe. The main strength is the nationwide coverage and accuracy of the register.

## Conclusion

In conclusion, the incidences of laboratory detected cases of *B. pertussis*,* C. pneumoniae*, and *M. pneumoniae* have been low throughout the pandemic period. Thus, it seems that the social restrictions have been effective in preventing the spreading of respiratory bacteria. How the ending of restrictions will affect to the detection rates should be addressed in the future.

## Supplementary Information

Below is the link to the electronic supplementary material.

Supplementary file1 (XLSX 25 KB)

## Data Availability

All data included as supplement.
